# A study on a tightening method for optimizing bolt preload distribution in high-precision assembly of curvic couplings for aerospace applications

**DOI:** 10.1371/journal.pone.0357915

**Published:** 2026-09-11

**Authors:** Jun Lin

**Affiliations:** School of Mechanical Engineering, Anhui University of Technology, Ma’anshan, Anhui, China; King Mongkut’s University of Technology North Bangkok, THAILAND

## Abstract

Non-uniform residual preload in bolt groups can reduce the assembly accuracy and interface-load uniformity of aerospace curvic-coupling structures. This study investigates residual-preload redistribution in a ten-bolt curvic-coupling assembly under different tightening strategies and initial preload levels. An equally sized shaft disk is used as a flat-interface reference structure to isolate the influence of contact-interface morphology from other geometric and material factors. Finite-element simulations are combined with an elastic-interaction framework to compare single-step, two-step, and three-step tightening paths. The results show that the periodic tooth-to-tooth interface of the curvic coupling changes local contact stiffness and load-transfer paths, thereby markedly amplifying bolt-to-bolt residual-preload differences compared with the shaft disk. Although the curvic-coupling structure does not change the basic role of tightening sequence in shaping the relative distribution pattern, it increases the sensitivity of residual preload to loading path and staged tightening. Among the investigated strategies, sequential tightening is not recommended, whereas the dual-axis crisscross strategy provides better preload uniformity. For high-precision assembly, the three-step dual-axis crisscross path of 40%–80%–100% provides the best overall uniformity under the present working conditions. The study clarifies why conclusions obtained from conventional flat-interface bolted joints cannot be directly transferred to curvic couplings and provides process-level guidance for tightening-path design in high-precision curvic-coupling assembly.

## Introduction

Bolted joints are widely used in aircraft engines and other mechanical structures [1]. The magnitude and consistency of preload directly affect assembly accuracy, in-service reliability, and the mechanical performance of the joint system [2–8]. During assembly, friction scatter, tightening sequence, staged loading, and bolt-group elastic interaction can cause residual preload to disperse and redistribute [9–15]. This redistribution changes the contact state and load-transfer path at the joint interface, and may further reduce fatigue strength, stiffness stability, and long-term service reliability [16–19]. Accordingly, understanding how the tightening path affects residual-preload distribution is of direct engineering importance for high-precision bolted assemblies.

Existing studies on bolt-group residual preload have mainly focused on common flat-interface structures such as flanges, casing joints, and shaft-disk connections [10,13–15,19,20]. These studies have shown that optimized tightening sequences and stepwise tightening can reduce bolt-load scatter by controlling elastic interaction among bolts. However, these conclusions were generally obtained from structures in which the contact interface is relatively continuous and the local contact stiffness varies more smoothly along the circumferential direction. In contrast, a curvic coupling transfers load through a periodic tooth-to-tooth contact interface. Previous studies on curvic couplings have mainly addressed contact stress, positioning accuracy, mounting error, transmission response, and rotor dynamic behavior [21–25]. These studies indicate that the contact interface is a dominant structural feature of curvic-coupling systems, but the influence of this periodic tooth-contact interface on bolt-group residual-preload redistribution under different tightening paths has not been sufficiently clarified.

This gap is important because uneven preload in a curvic-coupling bolt group may lead to non-uniform interface loading and altered contact conditions, increasing the risk of biased tooth-surface loading. Therefore, tightening strategies that are effective for flat-interface joints cannot be directly transferred to curvic-coupling assemblies without additional evaluation. To address this problem, the present study takes a curvic coupling and an equally sized shaft disk as comparative objects. The shaft disk serves as a baseline structure with a relatively uniform flat contact interface, whereas the curvic coupling is characterized by periodic tooth-to-tooth contact, non-uniform local contact stiffness, and different load-transfer paths. This comparison makes it possible to distinguish the effect of contact-interface morphology on residual-preload redistribution from other structural factors. Specifically, this study compares different tightening sequences, analyzes the influence of initial preload, and evaluates multi-step tightening profiles to identify a tightening path that improves preload uniformity in the curvic-coupling bolt group. The novelty of this work lies in connecting bolt-group elastic interaction with the contact-interface morphology of curvic couplings and in clarifying why process conclusions derived from flat-interface bolted joints should be re-evaluated for high-precision curvic-coupling assemblies.

## Methodology and theoretical analysis

### Theoretical model of residual bolt preload in bolt groups under elastic interaction

The elastic interaction of a bolt group refers to the influence of subsequent loading on the residual-preload state of bolts that have already been tightened during a sequential loading process. In essence, it reflects the redistribution of preload-induced stress and deformation within the joint system. To describe the evolution of residual preload under different tightening strategies, the linear relationship model proposed by Bibel and Ezell [26] is adopted in this study. Here, the model is used to interpret the preload-redistribution mechanism induced by different loading paths rather than to establish a complete closed-form analytical solution. Because the focus of this paper is the effect of curvic-coupling structure on residual-preload redistribution, the elastic interaction coefficient between bolts is taken as the key parameter in the theoretical analysis. The governing relationship is expressed as follows:


$$\begin{array}{c} \left\{F_{0}\right\}+[A]\left\{\Delta F_{f}\right\}=\left\{F_{f}\right\} \end{array}$$ (1)

where $\left\{F_{0}\right\}$ is the vector of bolt preloads before a given loading step (n×1), [*A*] is the elastic interaction coefficient matrix (n×n), *n* is the number of bolts in the bolt group, $\left\{\Delta F_{f}\right\}$ is the preload increment vector applied in the current loading step (n×1), and $\left\{F_{f}\right\}$ is the residual preload vector after completion of the current loading step (n×1).

The components of the elastic interaction coefficient matrix are written as:


$$\begin{array}{c} a_{ij}=\frac{f_{ij}-f_{i(j-1)}}{\Delta f_{j}} \end{array}$$ (2)

which quantify the coupling response of bolt *i* to the loading increment applied to bolt *j*. Specifically, $f_{ij}$ denotes the residual preload of bolt *i* after bolt *j* has been loaded, $f_{i(j-1)}$ denotes the residual preload of bolt *i* after completion of the previous loading step, and $\Delta f_{j}$ denotes the preload increment applied to bolt *j* in the current step. Accordingly, $a_{ij}$ represents the preload-response coefficient of bolt *i* per unit preload increment applied to bolt *j*.

On this basis, the elastic interaction framework is used to interpret the redistribution characteristics of residual preload under both single-step and multi-step tightening conditions. In the following analysis, a curvic coupling connected by ten bolts and an equally sized shaft disk are used as representative cases to compare the redistribution behavior of residual preload under different loading paths.

In the present study, the elastic-interaction model is used to interpret the redistribution mechanism rather than to provide a complete closed-form solution for the curvic-coupling system. According to Eq. (1), the residual preload after a given tightening step depends not only on the preload increment applied in that step, but also on the residual-preload state established in the preceding steps. Therefore, in two-step and three-step tightening, the subsequent loading operation should not be regarded as a simple superposition of additional preload. Instead, it redistributes the existing residual-preload state through the compliance of the bolt group and the joint interface. This interpretation provides the theoretical basis for explaining why staged tightening can reduce preload dispersion and why the curvic coupling, with its non-uniform tooth-contact interface, is more sensitive to the loading path than the flat-interface shaft disk.

### Simulation model of residual bolt preload in bolt groups

**Fig 1:**
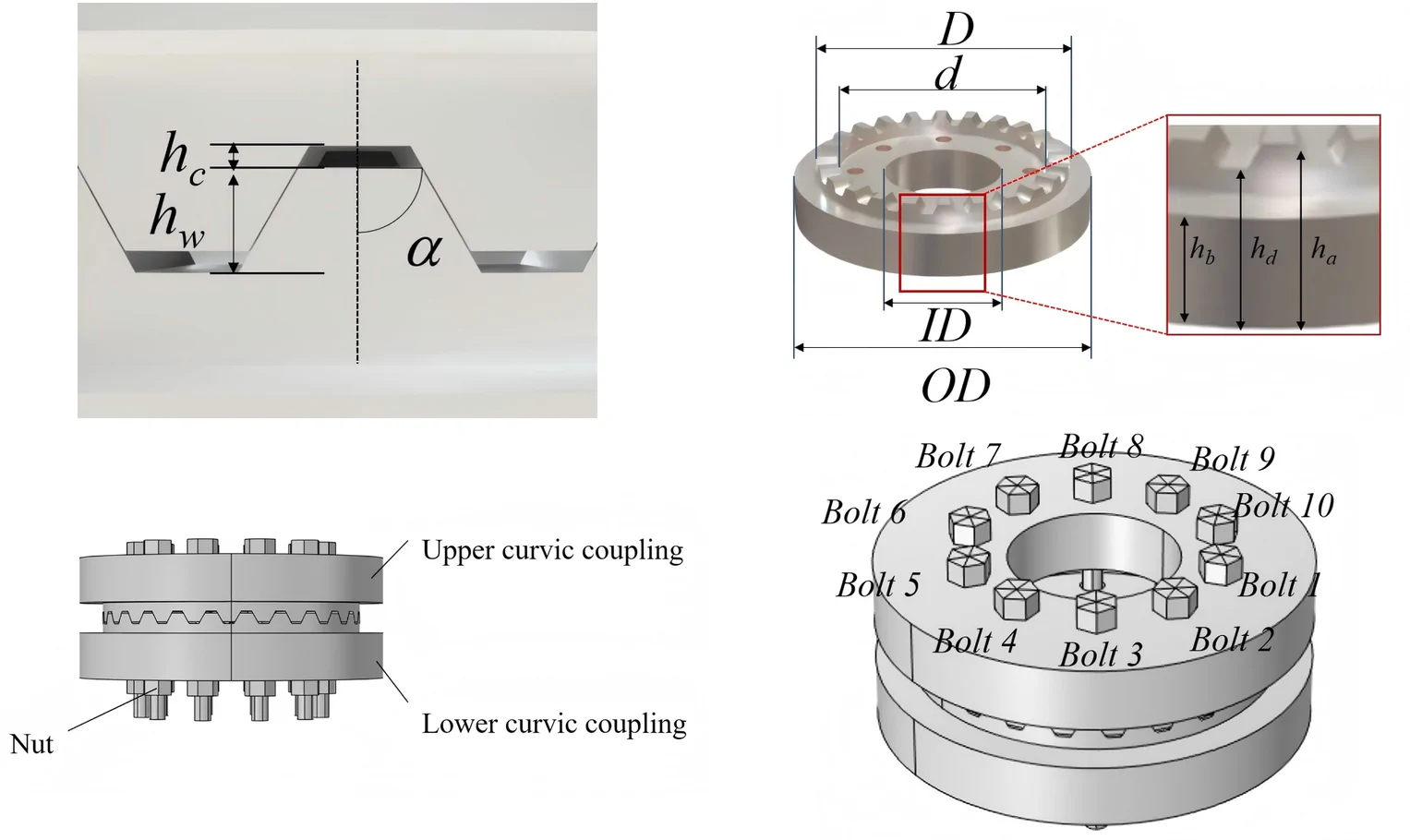
Curvic coupling model.

This study employs the commercial finite-element software COMSOL (Version 6.1) to analyze the effect of elastic interaction on the residual-preload distribution of bolt groups. Because the focus is the distribution pattern of residual preload rather than the torque-to-preload conversion process, preload is applied directly and thread details are not modeled, so that the influence of contact-interface configuration and loading path on bolt-group elastic interaction can be examined more clearly [27]. A shaft disk and a curvic coupling with the same material and overall dimensions are compared. The shaft disk is treated as a baseline structure with a relatively uniform flat contact interface, whereas the curvic coupling is characterized by periodic tooth-to-tooth contact, which introduces non-uniform local contact stiffness and load transfer paths. This comparison therefore makes it possible to examine the influence of contact-interface morphology on residual-preload redistribution under comparable geometric and material conditions. In all simulations, the contact-surface friction coefficient is set to 0.15, the connecting bolts are M10 grade 12.9 hex head bolts, and the curvic-coupling geometry is consistent with the preliminary design reported by our research group [21]. The dimensional specifications are listed in Table 1, and the corresponding model is shown in Fig 1.

**Table 1:** Dimensions of the curvic coupling.

Parameters	Symbol	Data	Parameters	Symbol	Data
Outer diameter of base (mm)	OD	152.4	Dedendum height (mm)	$h_{d}$	27
Inner diameter of base (mm)	ID	60	Tooth height (mm)	$h_{a}$	32.1
Outer diameter of ring gear (mm)	D	129	Base height (mm)	$h_{b}$	22.5
Inner diameter of face ring (mm)	d	108	Clearance (mm)	$h_{c}$	0.9
Angle of attack (°)	α	30	Total tooth height (mm)	$h_{w}$	5.1
Number of teeth	–	24			

**Fig 2:**
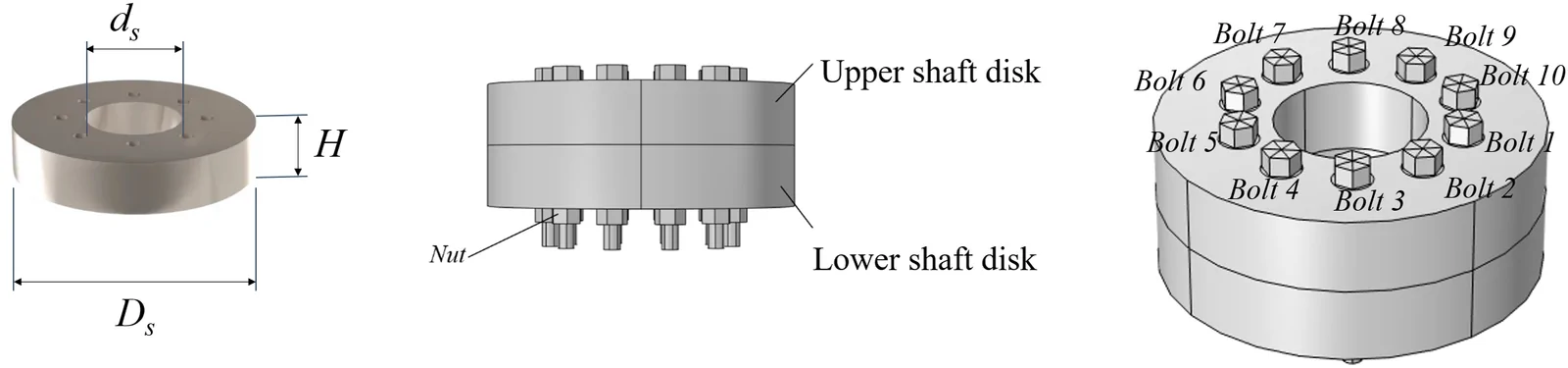
Shaft disk model.

To minimize the influence of non-target factors, the shaft disk used in this paper is designed with the same overall size as the curvic coupling. In this way, the main difference between the two comparative models is concentrated at the contact interface, which is beneficial for isolating the effect of interface morphology on preload redistribution. The dimensional parameters are shown in Table 2, and the model is shown in Fig 2.

**Table 2:** Shaft disk dimensions.

Parameters	Symbol	Data
Outer diameter (mm)	$D_{s}$	152.4
Inner diameter (mm)	$d_{s}$	60
Thickness (mm)	H	30

Pan et al. found that when the materials of the fastener and the workpiece are identical, using a material with a higher Poisson’s ratio and Young’s modulus can reduce the dispersion of residual preload [28]. Therefore, the material used in this paper is structural steel, with a Young’s modulus of 200 GPa, a Poisson’s ratio of 0.3, a density of 7850 kg/m^3^, and a yield strength of 350 MPa.

#### Model simplification and basic assumptions.

To focus on the redistribution characteristics of residual preload under different tightening paths, the finite-element model was established with the following simplifying assumptions. First, preload was applied directly to the bolt shank, and the torque-to-preload conversion process was not modeled. Accordingly, thread geometry, thread engagement, and torque-to-preload conversion scatter were not included. Second, the components were treated as homogeneous, isotropic, and linearly elastic, and material plasticity was not considered. Third, manufacturing error, tooth-profile deviation, assembly eccentricity, thermal loading, and service-speed effects were excluded. Fourth, the same material properties, bolt specifications, contact algorithm, friction setting, and boundary conditions were used for the curvic coupling and the shaft disk, with the contact-interface morphology being the intended difference between the two comparative models.

These simplifications were introduced to isolate the influence of tightening path and contact-interface morphology on residual-preload redistribution. In real torque-controlled assembly, friction, thread geometry, surface condition, and tightening-tool variability may affect the initial preload input. However, once preload has been introduced into the bolt group, the subsequent redistribution is governed by joint compliance, interface contact, and loading path. Therefore, the present model is intended to clarify the relative process-level influence of different tightening strategies under prescribed preload input, rather than to predict the full preload scatter of a complete torque-controlled tightening process.

#### Boundary conditions and constraint settings.

To ensure comparability between the curvic-coupling model and the shaft-disk model, all material parameters, geometric dimensions, bolt specifications, friction coefficients, and boundary conditions were kept identical except for the morphology of the contact interface. In the calculations, the lower curvic coupling or lower shaft disk was constrained against rigid-body motion to eliminate overall rigid-body displacement and rotation, whereas no additional displacement constraint was imposed on the upper component. Its response was therefore governed jointly by bolt preload and interface contact. Identity Boundary Pairs were used to represent the bolt–nut pair and the bolt/nut-to-component interfaces so that load transfer could remain continuous within the joint system. The preload of each bolt was applied to the same cross-sectional position of the bolt shank, and the loading direction was aligned with the bolt axis. In this way, differences among working conditions were controlled to arise only from tightening sequence and the number of tightening steps.

#### Contact relations, contact algorithm, and friction model.

Frictional contact was defined between the mating curvic-coupling surfaces and between the mating shaft-disk surfaces, and the coefficient of friction was uniformly set to 0.15. The normal contact constraint was enforced using the penalty method, while tangential behavior was described by the Coulomb friction model. In the curvic-coupling model, the periodic tooth-to-tooth interface served as the principal contact region; in the shaft-disk model, the corresponding flat interface served as the reference contact region. To avoid introducing additional differences through contact definition, the same contact algorithm, friction parameters, and solution controls were adopted for both models, with interface morphology being the only intended difference.

#### Preloading path and load-step settings.

To ensure stable closure of the contact interface before formal loading, all working conditions were initialized by applying a seating load equal to 1% of the target preload to all bolts. Formal loading was then carried out step by step according to the prescribed tightening sequence. For single-step tightening, each bolt was directly loaded to 100% of the target preload in the prescribed order. For two-step tightening, each bolt was first loaded, in the same sequence, to 20%, 40%, 60%, or 80% of the target preload and was then supplemented in the second stage until 100% was reached. For three-step tightening, the bolts were loaded sequentially according to the three-step profiles of 40%–70%–100%, 40%–80%–100%, and 50%–70%–100%, respectively. In all cases, subsequent loading was applied on the basis of the residual preload state established in the preceding step. The preload introduced in earlier steps was maintained during later steps so as to reproduce the cumulative nature of staged tightening in practical assembly.

#### Meshing strategy.

A mixed mesh was adopted for discretization. Because the bolt shanks and the contact-interface region are more sensitive to the residual-preload distribution, the bolt shanks were refined relative to the remaining components, whereas the other regions were meshed more coarsely to balance accuracy and computational efficiency. The final curvic-coupling model contained 78,165 domain elements, 23,122 boundary elements, and 6,001 edge elements, while the shaft-disk model contained 92,196 domain elements, 22,116 boundary elements, and 5,402 edge elements. The same meshing principles and nominal refinement level were used for both models to preserve comparability of the numerical results.

#### Solver settings and nonlinear solution parameters.

Because the model involved frictional contact and staged loading, the calculations were performed as a nonlinear static analysis. A direct solver was employed together with automatic load stepping to improve the stability of the contact solution. Convergence was monitored using the relative tolerance specified in the solver settings. Material plasticity was not considered, so all components retained linear-elastic constitutive behavior. However, because the contact interfaces underwent closure and frictional interaction during loading, the model included contact nonlinearity.

#### Data extraction and statistical procedure.

Residual-preload data were extracted from the COMSOL results module. The extraction position was kept identical for all bolts and corresponded to the preload-defined cross-section of the bolt shank. For loading-path analysis, after each individual bolt had been tightened and the system had reached equilibrium, the preload changes of all bolts were extracted to track the evolution of residual preload during the tightening process. For statistical analysis, the final equilibrium-state data after completion of all loading steps were used uniformly to calculate the mean value, minimum value, standard deviation, and normalized residual-preload ratio. In the present study, the standard deviation was taken as the principal indicator of preload dispersion, whereas the normalized residual-preload ratio was used to characterize the relative position and variation trend of each bolt within the bolt group.

### Tightening sequence and tightening strategy

**Fig 3:**
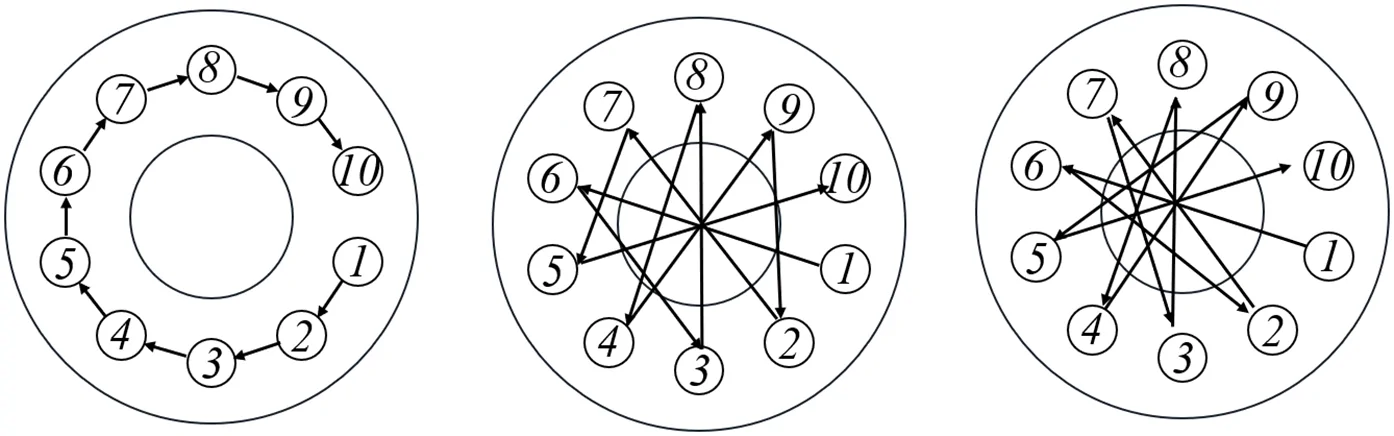
The tightening sequences used. Specifically, the sequential tightening sequence is 1–2–3–4–5–6–7–8–9–10. The tightening sequence for single-axis crisscross tightening is 1–6–3–8–4–9–2–7–5–10. The tightening sequence for dual-axis crisscross tightening is 1–6–2–7–3–8–4–9–5–10.

The ASME PCC-1–2019 Guidelines recommend several tightening-sequence layouts for bolted flange assemblies, including single-axis, dual-axis, and four-axis arrangements. Sequential tightening is the simplest method, but it is also more likely to amplify elastic interaction and thus aggravate off-center loading [29]. Single-axis crisscross tightening is a representative approach and performs well in single-step preloading. To further reduce preload variation, aircraft-engine manufacturers have also adopted a dual-axis crisscross tightening strategy in recent years, as shown in Fig 3.

### Methods for measuring the distribution of residual preload

To characterize the residual-preload distribution of the bolt group from different perspectives, the mean value, minimum value, standard deviation, and normalized residual-preload ratio were adopted as evaluation indices. The mean value reflects the overall residual-preload level of the bolt group, the minimum value represents the lowest residual preload obtained under a given tightening process and thus reflects preload loss, and the standard deviation quantifies the overall dispersion of residual preload within the bolt group. To compare the relative position of each bolt within the group under different tightening strategies, a normalized residual-preload ratio was introduced as an auxiliary indicator.

The normalized residual preload ratio is defined by Eq (3):


$$\begin{array}{c} \xi =\frac{x_{i}}{x_{0}} \end{array}$$ (3)

where $x_{i}$ denotes the residual preload of the *i*-th bolt and *x*_0_ denotes the median residual preload of the bolt group under the corresponding working condition. The normalized residual-preload ratio is therefore used to characterize the relative distribution feature and variation range of residual preload within the bolt group, whereas the standard deviation is retained as the principal statistical indicator of overall dispersion.

### Model validation plan and scope of applicability

A dedicated physical validation experiment is not available at the present stage. Therefore, the present finite-element results should be interpreted within a clearly defined applicability boundary. The simulations describe a ten-bolt, twenty-four-tooth curvic-coupling assembly and an equally sized shaft disk under prescribed preload input, structural-steel material properties, static loading, idealized geometry, and frictional contact conditions. The results mainly reflect the relative influence of tightening path and contact-interface morphology on residual-preload redistribution, rather than the complete preload scatter in a real torque-controlled assembly process.

Future validation will be carried out in three steps. First, representative tightening cases will be compared with the elastic-interaction framework to check the consistency between the finite-element redistribution trend and the theoretical preload-response mechanism. Second, a curvic-coupling test rig and a reference shaft-disk assembly will be fabricated. Bolt residual preload will be measured using instrumented bolts, ultrasonic preload assessment, or strain gauges, and the interface pressure distribution will be examined using pressure-sensitive films when feasible. Third, the measured mean residual preload, minimum residual preload, standard deviation, and normalized residual-preload ratio will be compared with the corresponding finite-element predictions. This validation route will allow the numerical results to be further assessed without extending the present paper beyond its simulation-based scope.

## Results and discussion

### Differences in preload distribution between the curvic coupling and the shaft disk under single-step tightening

**Fig 4:**
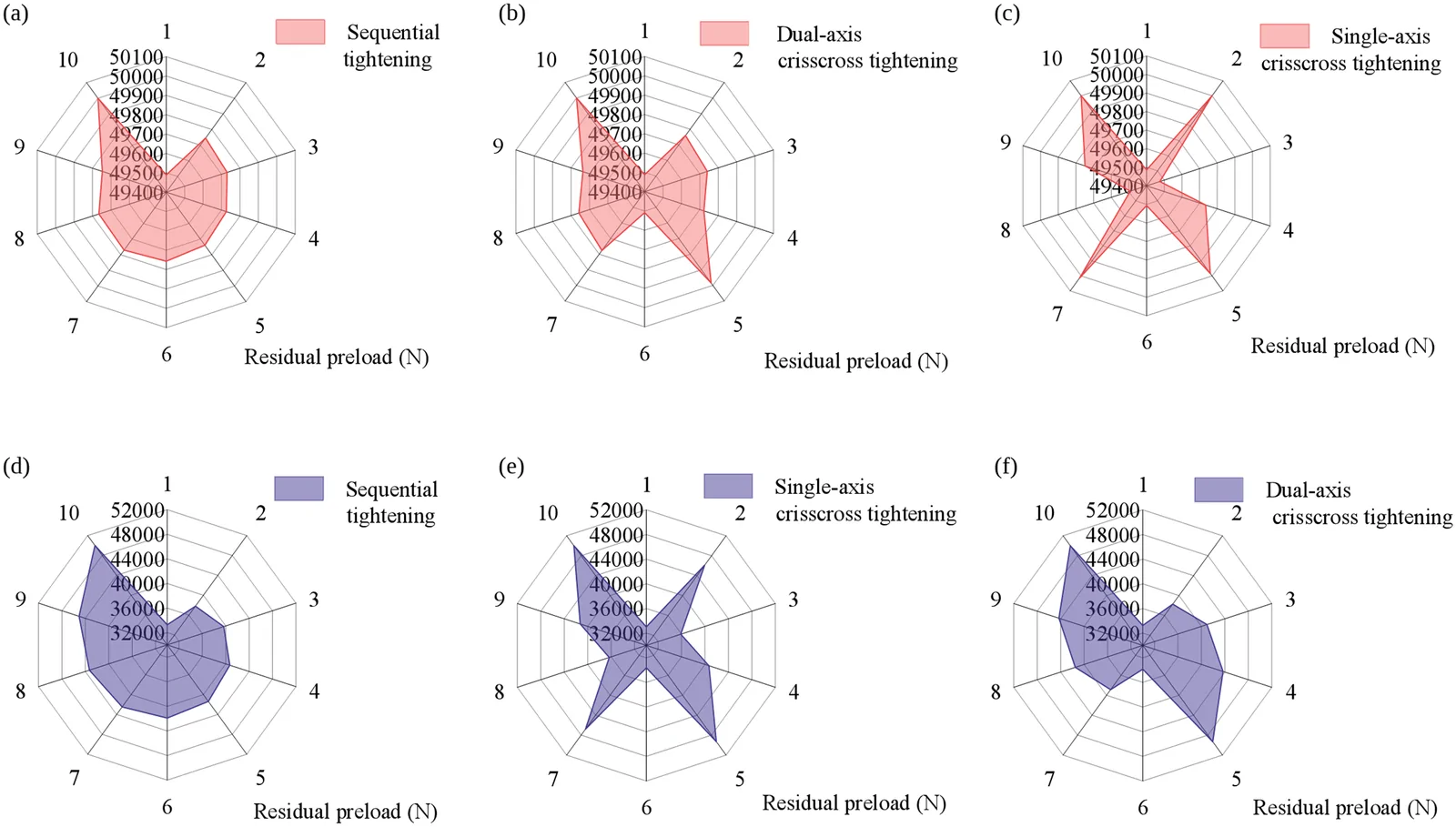
Distribution of residual preload after single-step tightening. (a) Sequential tightening of the shaft disks. (b) Dual-axis crisscross tightening of the shaft disks. (c) Single-axis crisscross tightening of the shaft disks. (d) Sequential tightening of the curvic couplings. (e) Single-axis crisscross tightening of the curvic couplings. (f) Dual-axis crisscross tightening of the curvic couplings.

Based on the preceding analysis, this section uses single-step tightening to compare the residual-preload distribution of the curvic-coupling and shaft-disk bolt groups under different tightening sequences, with the aim of identifying the influence of the curvic-coupling structure on preloading performance. During loading, the initial preload of each bolt was set to 50 kN [27]. The distributions of residual preload under the three tightening methods are shown in Fig 4, and the corresponding mean value, minimum value, and standard deviation are listed in Table 3. Here, the mean value reflects the overall preload level, the minimum value reflects the extent of preload loss, and the standard deviation reflects the overall degree of residual-preload dispersion. In addition, the normalized residual-preload ratio is used to describe the relative position and variation pattern of each bolt within the bolt group under different tightening methods.

**Table 3:** Coefficients of residual bolt preload for bolt groups under various methods.

Tightening method	Avg. residual preload (kN)	Min. residual preload (kN)	Std. deviation
Sequential tightening of the shaft disk	49.75	49.49	121.4
Single-axis crisscross tightening on the shaft disk	49.74	49.49	240.5
Dual-axis crisscross tightening on the shaft disk	49.75	49.49	165.3
Sequential tightening of the curvic coupling	41.56	33.28	4406.8
Single-axis crisscross with curvic coupling	41.30	33.02	6455.6
Dual-axis crisscross with curvic couplings	41.40	33.24	5677.3

Under single-step tightening, the shaft disk and the curvic coupling exhibit similar overall distribution trends of residual preload, but their numerical levels differ substantially. Taking bolts 10 and 5 as examples, the residual-preload difference in the curvic coupling is 32.07 times that in the shaft disk. This indicates that, although the basic action of the tightening sequence is similar in the two structures, differences in contact area and contact configuration cause the curvic-coupling structure to alter the load-transfer path and thereby markedly amplify bolt-to-bolt preload variation.

**Fig 5:**
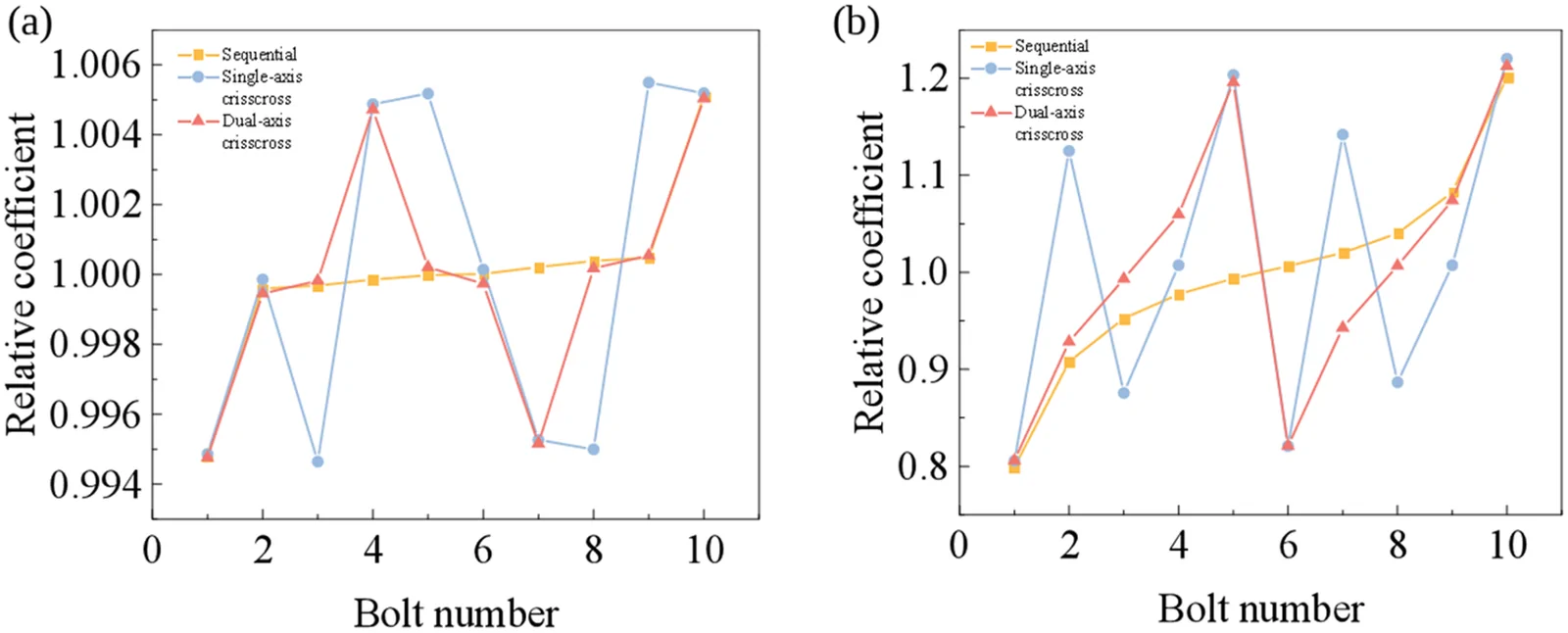
Normalized residual preload ratio of each bolt after single-step tightening. (a) Shaft disk. (b) Curvic coupling.

As shown by the normalized residual-preload ratio in Fig 5, the preload difference between adjacent bolts on the curvic coupling is significantly larger than that on the shaft disk, indicating that the curvic-coupling structure markedly amplifies local variation in residual preload within the bolt group. Under sequential tightening, this amplification is particularly pronounced at the edge bolts; the large gap between bolts 10 and 5 can readily increase the risk of biased loading during assembly, which is unfavorable for high-precision curvic-coupling assembly.

As shown in Table 3, under the same initial preload and tightening method, both the mean residual preload and the minimum residual preload of the curvic coupling are generally lower than those of the shaft disk, indicating that the curvic-coupling structure aggravates preload loss. At the same time, because the curvic-coupling structure strongly amplifies dispersion, the standard deviation of residual preload in the curvic coupling is 30.4 times that in the shaft disk, indicating much higher sensitivity to preload uniformity.

Taken together, these results show that the curvic-coupling structure does not alter the basic role of tightening strategy in shaping the overall residual-preload pattern, but it clearly enlarges the differences among bolts. Consequently, the same tightening strategy produces substantially different assembly performance in the curvic coupling and the shaft disk. This observation is consistent with previous studies showing that tightening sequence strongly affects bolt-load scatter and that optimized loading paths can improve preload uniformity in multi-bolt joints [13,14,20,30]. For curvic couplings, the single-axis and dual-axis crisscross strategies produce similar distribution trends; however, the dual-axis crisscross strategy reduces the standard deviation of residual preload by 12.06% relative to the single-axis crisscross strategy. Therefore, for curvic-coupling assembly, the dual-axis crisscross strategy is preferable, whereas sequential tightening should be avoided.

### Differences in preload distribution between the curvic coupling and shaft disk under different initial preloads in a single-step tightening operation

**Fig 6:**
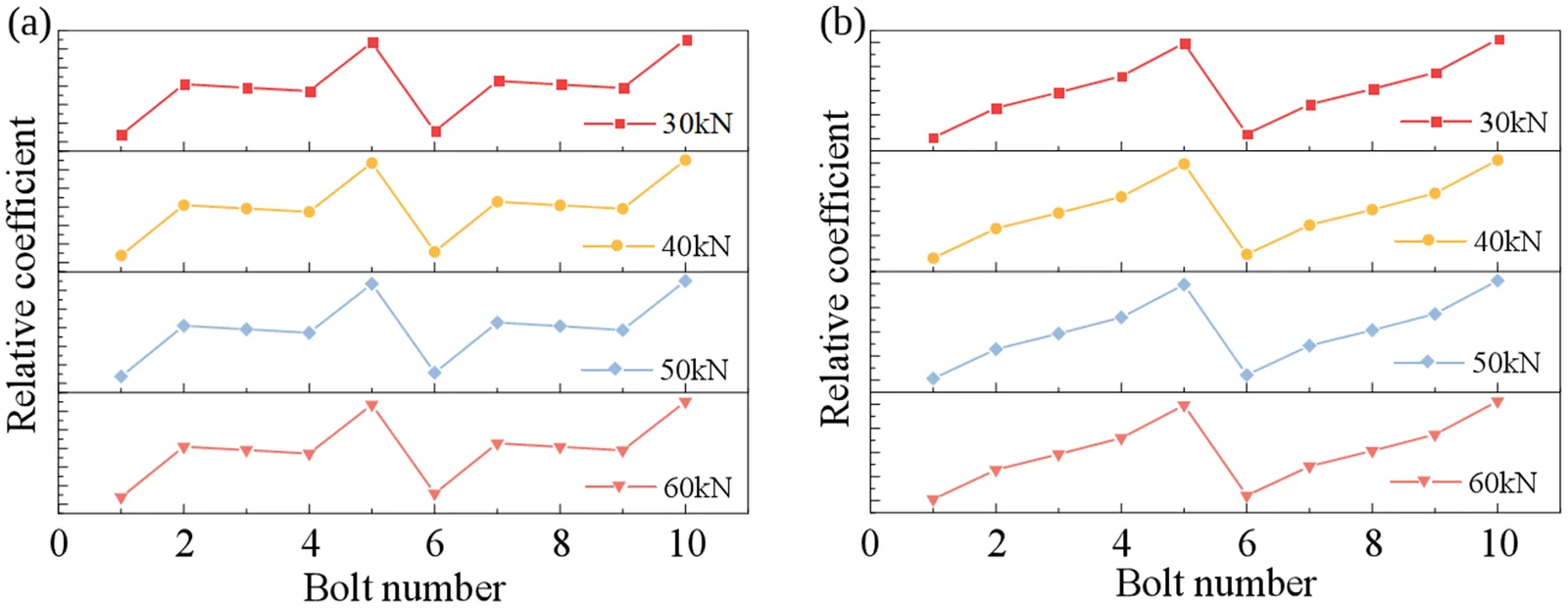
Normalized residual preload ratio of each bolt under different initial preload conditions. (a) Shaft disk. (b) Curvic coupling.

Among the three tightening methods examined in the previous section, the dual-axis crisscross strategy provides the best single-step preloading performance. To further investigate the effect of initial preload on the residual-preload distribution of curvic-coupling bolt groups, this section compares four initial preload levels—30 kN, 40 kN, 50 kN, and 60 kN—under the dual-axis crisscross strategy [27]. The normalized residual-preload ratio of each bolt under different initial-preload conditions is shown in Fig 6, and the corresponding mean value, minimum value, and standard deviation of residual preload are listed in Table 4.

**Table 4:** Coefficients of residual bolt preload for bolt groups under various initial preload conditions.

Initial preload	Avg. residual preload (kN)	Min. residual preload (kN)	Std. deviation
Shaft disk 30 kN	29.849	29.695	98.8
Shaft disk 40 kN	39.798	39.591	132.2
Shaft disk 50 kN	49.747	49.489	165.3
Shaft disk 60 kN	59.697	59.386	198.4
Curvic coupling 30 kN	24.835	19.931	3409.7
Curvic coupling 40 kN	33.119	26.582	4544.0
Curvic coupling 50 kN	41.402	33.237	5677.3
Curvic coupling 60 kN	49.687	39.893	6810.0

**Fig 7:**
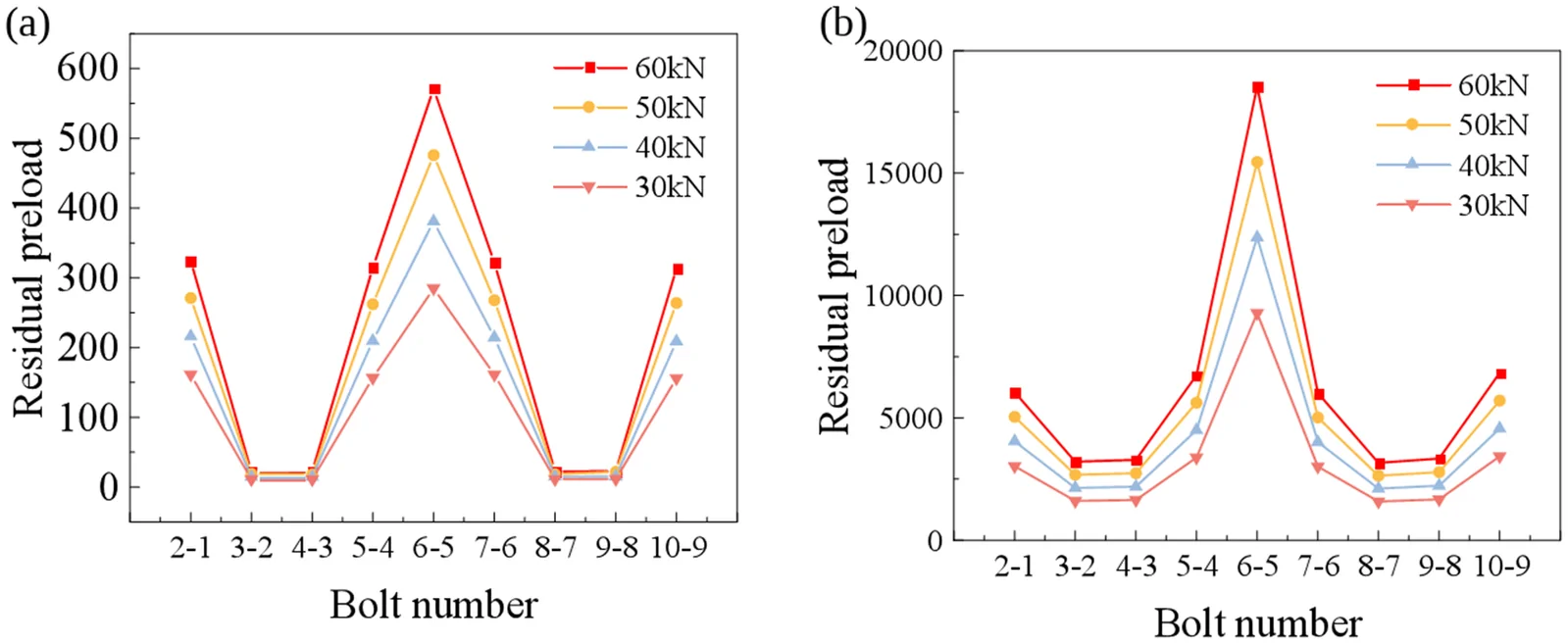
Differences in preload between adjacent bolts under different initial preloads. (a) Shaft disk. (b) Curvic coupling.

Combining Fig 6 with Table 4 shows that variation in the initial preload does not change the relative distribution pattern of residual preload in the bolt group, but mainly affects its magnitude. As the initial preload increases, the overall residual preload level of the bolt group also increases. This result is consistent with the elastic-interaction interpretation: under the same loading order, the preload-response path of the bolt group remains similar, so the normalized residual-preload ratio retains a comparable shape. However, because the curvic-coupling interface introduces non-uniform local contact stiffness and uneven load-transfer paths, a higher initial preload magnifies the absolute bolt-to-bolt difference even when the relative distribution pattern remains similar. From an assembly perspective, the preload level should therefore be selected by considering not only clamping capacity, but also assembly accuracy, allowable biased loading, and process operability. The preload differences between adjacent bolts under the four initial preload conditions are compared in Fig 7.

### Differences in preload distribution between the curvic coupling and the shaft disk under two-step tightening

**Fig 8:**
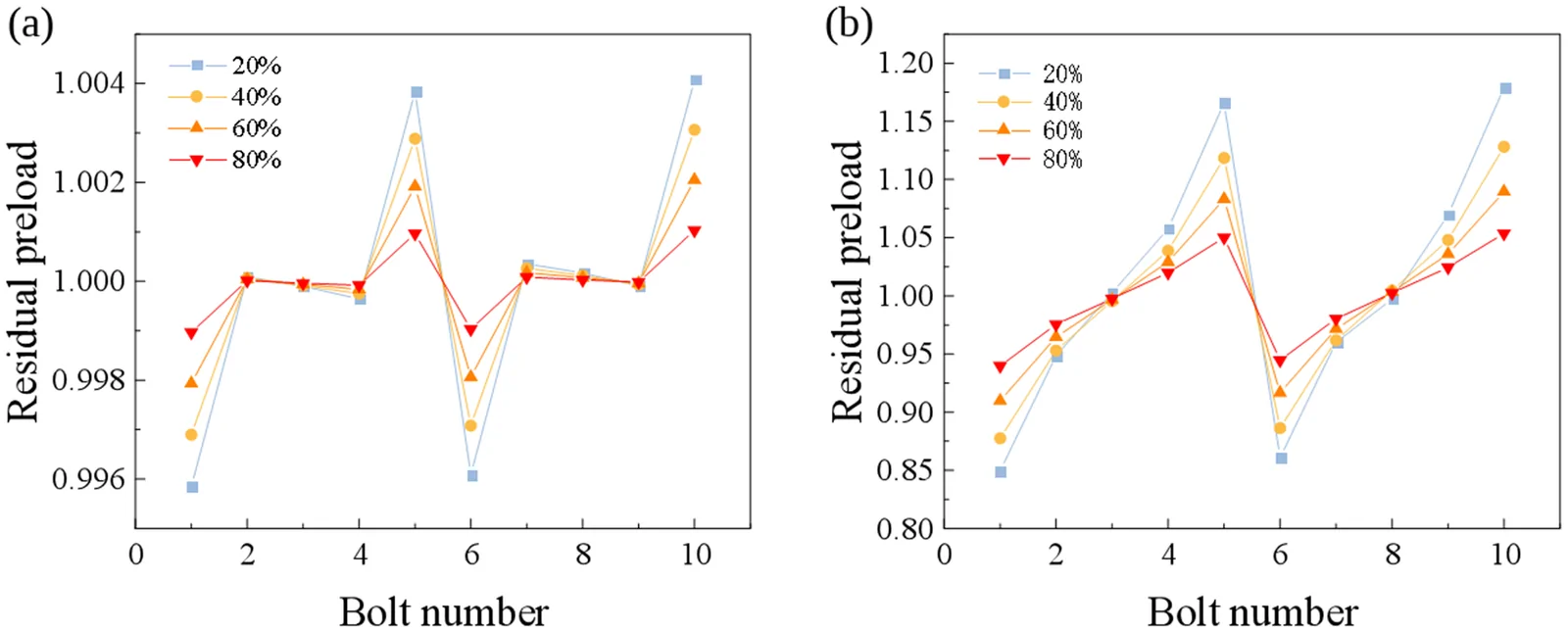
Normalized residual preload ratio of each bolt after two-step tightening. (a) Shaft disk. (b) Curvic coupling.

During two-step tightening, excessive preload in a single loading stage can increase elastic interaction between bolts and aggravate the non-uniformity of residual preload, whereas staged loading can promote preload redistribution and improve final uniformity. In this section, a second tightening stage is introduced after the initial loading stage to investigate the evolution of residual preload in the bolt groups of the curvic coupling and the shaft disk. The target preload is 50 kN for all cases. In the first loading stage, 20%, 40%, 60%, and 80% of the target preload are applied, respectively, and in the second loading stage the preload is supplemented to 50 kN. The mean value, minimum value, and standard deviation of the residual preload under the different two-step tightening conditions are summarized in Table 5, and the corresponding normalized residual-preload ratios are shown in Fig 8.

**Table 5:** Coefficients of residual bolt preload for bolt groups under various two-step tightening methods.

Two-step tightening method	Avg. residual preload (kN)	Min. residual preload (kN)	Std. deviation
Shaft disk 20%–100%	49.80	49.60	132.87
Curvic coupling 20%–100%	42.80	36.04	4753.62
Shaft disk 40%–100%	49.85	49.69	99.70
Curvic coupling 40%–100%	44.38	38.89	3792.44
Shaft disk 60%–100%	49.90	49.79	66.45
Curvic coupling 60%–100%	45.89	41.75	2831.99
Shaft disk 80%–100%	49.95	49.90	33.38
Curvic coupling 80%–100%	47.41	44.60	1875.35

Two-step tightening essentially decomposes the single-step tightening process into two loading stages. For a given tightening strategy, the second stage is not independent of the first; rather, it is applied to the residual-preload state established in the first stage. As indicated by Eq (1), the residual preload formed in the previous stage directly affects the subsequent preload response of the bolt group. The second-stage tightening should therefore be understood as a redistribution of the existing preload state rather than a simple superposition of load. This interpretation is consistent with previous analytical and predictive studies showing that elastic interaction governs bolt-to-bolt tension change during sequential loading and that staged tightening modifies residual preload primarily through redistribution [15,20]. The dependence of both dispersion and normalized residual-preload ratio on the second-stage increment therefore supports the physical interpretability of the present numerical results.

Stepwise tightening modifies the final residual-preload distribution because each subsequent loading step acts on the residual-preload state produced by the preceding step. The second stage is therefore a redistribution process rather than an independent loading operation. When the later-stage supplementary increment is smaller, the additional elastic interaction induced by that increment is also reduced, which helps narrow the variation range of the normalized residual-preload ratio. This mechanism explains why a higher preload proportion in the earlier stage and a smaller compensatory increment in the later stage are generally favorable for improving final preload uniformity.

Compared with the shaft disk, the curvic coupling still exhibits greater residual-preload dispersion under two-step tightening. As established in the previous section, the relative distribution of residual preload in the first loading stage is not affected by the magnitude of the initial preload under the same tightening strategy. The differences observed after two-step tightening therefore arise mainly from the second loading step. The smaller the supplementary preload applied in the second stage, the narrower the variation range of the normalized residual-preload ratio and the smaller the standard deviation of residual preload, indicating that a higher first-stage preload proportion is more favorable for final uniformity. For the shaft disk, two-step tightening mainly affects the outermost bolts on both sides because these bolts correspond to the first and last loading positions. By contrast, the residual preload of most bolts in the curvic coupling varies with the proportion of the second loading step, and the variation trend is generally consistent across all working conditions. This indicates that the curvic-coupling structure makes the overall distribution characteristics more sensitive to the second loading step. Compared with single-step tightening, two-step tightening provides better preload uniformity in curvic-coupling assembly. The results also suggest that a higher preload proportion in the first stage helps reduce the additional dispersion introduced during the second-stage supplementary loading.

### Differences in preload distribution between the curvic coupling and the shaft disk under three-step tightening

The preceding results show that stepwise tightening can significantly reduce the standard deviation of residual preload in the bolt group and that the second loading stage plays a dominant role in preload redistribution, with the curvic coupling showing greater sensitivity to this stage. To further improve assembly accuracy and preload uniformity, this section examines three-step tightening for the curvic coupling and the shaft disk with a target preload of 50 kN. Considering the practical difficulty of applying very low preload levels during assembly, three loading paths—40%–70%–100%, 40%–80%–100%, and 50%–70%–100%—are selected for comparison. The mean value, minimum value, and standard deviation of residual preload under the different tightening methods are listed in Table 6.

**Table 6:** Coefficients of residual bolt preload for bolt groups under various three-step tightening methods.

Part	Tightening method	Avg. residual (kN)	Min. residual (kN)	Std. deviation
Shaft disk	40%–70%–100%	49.93	49.85	44.6
Curvic coupling	40%–70%–100%	47.03	44.06	2032.2
Shaft disk	40%–80%–100%	49.95	49.90	29.7
Curvic coupling	40%–80%–100%	47.78	45.49	1553.4
Shaft disk	50%–70%–100%	49.93	49.85	44.6
Curvic coupling	50%–70%–100%	47.13	44.29	1951.3

**Fig 9:**
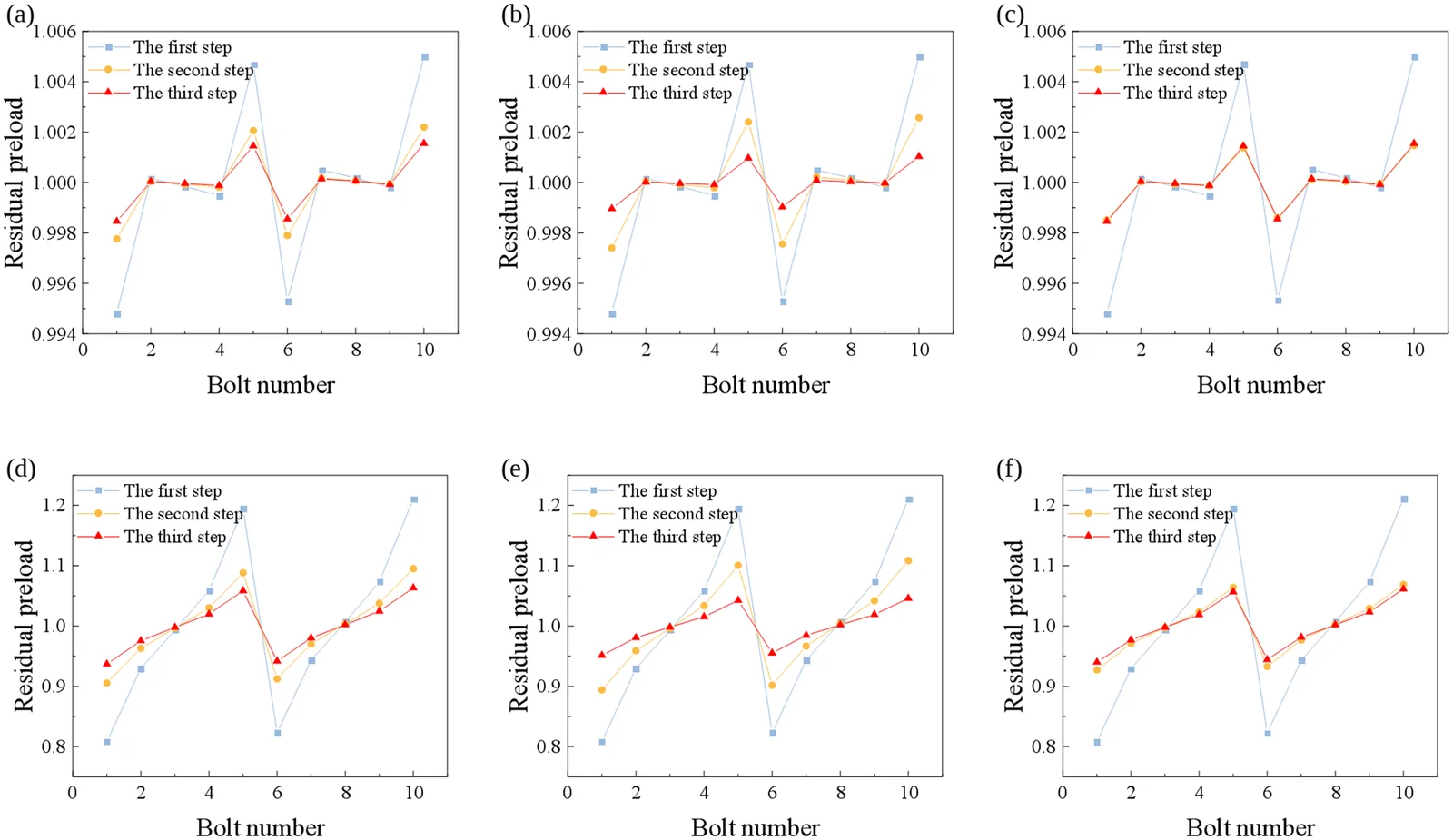
Normalized residual preload ratio of each bolt after three-step tightening. (a) Shaft disk 40%–70%–100%. (b) Shaft disk 40%–80%–100%. (c) Shaft disk 50%–70%–100%. (d) Curvic coupling 40%–70%–100%. (e) Curvic coupling 40%–80%–100%. (f) Curvic coupling 50%–70%–100%.

Under three-step tightening, the third loading stage redistributes the residual-preload state established after the first two stages and therefore plays a key role in refining the final preload distribution. This behavior is associated with the marked variation in contact stiffness and load-transfer paths at the tooth-contact interface. As the clamping load increases, the contact state becomes more sensitive to load variation, so the final loading stage triggers a stronger redistribution among bolts. For the three loading paths of 40%–70%–100%, 40%–80%–100%, and 50%–70%–100%, the standard deviation of residual preload in the curvic coupling is 44.56, 51.3, and 42.75 times that in the shaft disk, respectively. Thus, even under multi-step tightening, the curvic-coupling structure still markedly amplifies preload dispersion. The corresponding distributions of the normalized residual-preload ratio are shown in Fig 9.

The preceding results show that the amplifying effect of the curvic-coupling structure on residual-preload dispersion remains evident even when the tightening strategy or the initial preload is changed. For curvic couplings assembled using the coaxial tightening path, residual preload on the same side increases progressively with tightening sequence, whereas a sharper variation appears at the edge-bolt locations. Compared with the shaft disk of the same size, this edge-region variation is much larger in the curvic coupling. This indicates that the periodic tooth-to-tooth contact interface strengthens the boundary effect of load transfer by introducing non-uniform local constraints and equivalent contact stiffness, thereby amplifying elastic interaction among edge bolts. The resulting redistribution pattern is directly relevant to assembly uniformity and provides an engineering indication that curvic-coupling-based systems may be more sensitive to interface-related dynamic effects. This implication is not directly calculated in the present study, but it is consistent with previous studies that have reported strong sensitivity of curvic-coupling-based rotor systems to interface-related vibration characteristics [17,24,25]. Nevertheless, although the amplification effect remains present throughout all loading paths, three-step tightening still produces a more uniform final distribution than two-step tightening. Among the three-step schemes considered here, the 40%–80%–100% path yields the smallest standard deviation of residual preload and the best overall uniformity in the curvic coupling. Compared with single-step tightening, this scheme reduces the variation range of the normalized residual-preload ratio by 81.6% and the standard deviation of residual preload by 72.6%; compared with the 80%–100% two-step scheme that gives the smallest standard deviation among the two-step cases, the corresponding additional reductions are 16.59% and 17.17%, respectively. These results indicate that three-step tightening can further improve the residual-preload distribution of the curvic-coupling bolt group, although the gain relative to two-step tightening is smaller than that relative to single-step tightening. Therefore, the 40%–80%–100% path should be recommended mainly for high-precision assembly scenarios in which preload uniformity is the dominant requirement. For general-precision or efficiency-oriented assembly, the additional tightening pass, assembly time, operational complexity, and diminishing improvement should be considered together.

The present results show that the curvic-coupling structure amplifies residual-preload dispersion not because the basic influence of tightening sequence is altered, but because the periodic tooth-to-tooth contact interface introduces non-uniform local contact stiffness and load-transfer paths. Under otherwise comparable geometric and material conditions, this interface morphology increases the sensitivity of edge bolts to elastic interaction and strengthens the path dependence of preload redistribution. Consequently, the same tightening strategy produces markedly different preload-distribution outcomes in the curvic coupling and the shaft disk.

From the perspective of existing bolted-joint literature, the present findings are broadly consistent with previous studies on flat-interface structures such as flanges and shaft disks, in that tightening sequence and staged loading still govern the overall redistribution trend of residual preload [13–15,20]. However, the amplification effect observed in the curvic coupling is more pronounced, indicating that conclusions derived from common flat-interface joints cannot be directly transferred to curvic-coupling assemblies without considering the non-uniformity of the tooth-contact interface. This distinction constitutes the principal contribution of the present study relative to conventional bolt-group preload analysis.

These results show that the choice of tightening path has a direct influence on the assembly quality of the curvic coupling. Sequential tightening is unfavorable because it enlarges preload differences, especially at the edge bolts, and therefore increases the likelihood of biased tooth-surface loading. Under single-step tightening, the dual-axis crisscross strategy provides a more uniform residual preload distribution than the other loading paths considered in this study. The results further show that staged tightening is effective in suppressing preload dispersion. In particular, a relatively higher preload proportion in the earlier stage, followed by a smaller compensatory increment, is more beneficial to assembly uniformity. This interpretation is also in line with previous studies showing that preload level and preload asymmetry can strongly affect the durability and dynamic behavior of aerospace fastened joints and curvic-coupling-based rotor systems [5,17,20,24].

Several limitations of the present study should also be acknowledged. The model does not include thread-level details or torque-to-preload scatter, and manufacturing error, assembly eccentricity, thermal loading, and service-speed effects are not considered. In addition, the conclusions are derived from finite-element simulations under a specific set of geometric, material, and contact conditions, and no dedicated experimental validation has yet been incorporated. Future work should therefore extend the present analysis through experimental validation, interface-related output variables, torque-to-preload scatter modeling, and broader parametric studies involving friction coefficient, material stiffness, manufacturing error, assembly eccentricity, thermal loading, and service-speed effects.

## Conclusion

This study used finite-element simulation and an elastic-interaction framework to investigate residual-preload redistribution in a ten-bolt curvic-coupling assembly. An equally sized shaft disk was used as a flat-interface reference structure so that the influence of contact-interface morphology could be distinguished from other geometric and material factors. The main conclusions are as follows.

First, the curvic-coupling structure markedly amplifies residual-preload dispersion compared with the shaft disk. This amplification is not caused by a change in the basic action of the tightening sequence, but by the periodic tooth-to-tooth contact interface, which introduces non-uniform local contact stiffness and different load-transfer paths. Therefore, tightening conclusions obtained from conventional flat-interface bolted joints should not be directly transferred to curvic-coupling assemblies without considering interface morphology.

Second, under single-step tightening, sequential tightening should be avoided for curvic-coupling assembly because it enlarges bolt-to-bolt preload differences and increases the risk of non-uniform interface loading. The dual-axis crisscross strategy provides better preload uniformity under the present working conditions and is therefore more suitable as the basic tightening path for the curvic-coupling bolt group.

Third, changing the initial preload mainly changes the absolute residual-preload level rather than the relative distribution pattern. However, because the curvic-coupling interface amplifies local stiffness differences, a higher initial preload also enlarges the absolute bolt-to-bolt residual-preload difference. The preload level should therefore be selected by balancing clamping capacity, assembly accuracy, biased-loading risk, and process operability.

Fourth, staged tightening improves preload uniformity because subsequent loading redistributes the residual-preload state formed in the preceding step rather than simply adding a new independent load. Among the loading paths examined in this study, the 40%–80%–100% three-step dual-axis crisscross path provides the best preload uniformity for the curvic coupling. However, its improvement over the best two-step path is incremental, so the choice between two-step and three-step tightening should consider assembly accuracy requirements, tightening time, operational complexity, and diminishing returns.

The present results should be understood as process-level findings under idealized geometry, prescribed preload input, static loading, and the specified material and contact conditions. Future work will focus on experimental validation using bolt-force measurement, ultrasonic preload assessment, strain gauges, and interface pressure measurements. Further studies should also incorporate torque-to-preload scatter, thread geometry, friction and material-parameter sensitivity, manufacturing error, assembly eccentricity, thermal loading, service-speed effects, contact pressure, tooth-surface load distribution, and dynamic response of curvic-coupling systems.

## Supporting information

S1 Data Minimal dataset underlying the findings of this study. This supporting file provides the minimal dataset required to replicate all findings reported in this article. All data required to replicate the reported means, minimum values, standard deviations, normalized residual-preload ratios, adjacent-bolt preload differences, figures, tables, and conclusions are provided in the manuscript and in S1_Data.xlsx. S1_Data.xlsx contains bolt-level residual-preload values, figure source data, summary statistics, friction-coefficient verification data, mesh-independence verification data, model parameters, and English-translated step-by-step residual-preload matrices for variable initial-preload, single-step, two-step, and three-step tightening conditions.
(XLSX)

S1 Appendix Minimal dataset summaries. This appendix contains Appendix Tables A1–A7, including model parameters and tightening paths, bolt-level residual preload values for single-step tightening, different initial-preload levels, two-step tightening, and three-step tightening, as well as friction-coefficient verification and mesh-independence verification results. (DOCX)
