# Supplementary material for: A study on a tightening method for optimizing bolt preload distribution in high-precision assembly of curvic couplings for aerospace applications
Source: PLoS One. 2026 Sep 11;21(9):e0357915. doi: 10.1371/journal.pone.0357915 (PMC13567825; doi:10.1371/journal.pone.0357915)
Supplement: Supplementary file 1 [file pone.0357915.s002.docx]

Appendix A. Minimal dataset summaries

The following appendix summarizes the numerical data underlying the main tables and figures in the manuscript. Bolt-level residual preload values are reported in N unless otherwise stated. A complete spreadsheet version of the dataset is also provided as S1 Data, including English-translated step-by-step residual-preload matrices for the variable initial-preload, single-step, two-step, and three-step tightening cases.

**Appendix Table A1. Model parameters and tightening paths.**

| Category | Parameter | Value | Unit/Description |
| --- | --- | --- | --- |
| Material | Material type | Structural steel | Used for bolts, curvic coupling, and shaft disk |
| Material | Young’s modulus | 200 | GPa |
| Material | Poisson’s ratio | 0.3 | - |
| Material | Density | 7850 | kg/m3 |
| Material | Yield strength | 350 | MPa |
| Contact | Friction coefficient in main simulations | 0.15 | - |
| Bolt | Bolt specification | M10 grade 12.9 hex head bolts | - |
| Geometry | Number of bolts | 10 | - |
| Geometry | Number of curvic-coupling teeth | 24 | - |
| Tightening sequence | Sequential tightening | 1-2-3-4-5-6-7-8-9-10 | Bolt order |
| Tightening sequence | Single-axis crisscross tightening | 1-6-3-8-4-9-2-7-5-10 | Bolt order |
| Tightening sequence | Dual-axis crisscross tightening | 1-6-2-7-3-8-4-9-5-10 | Bolt order |
| Two-step path | Two-step tightening | 20%-100%; 40%-100%; 60%-100%; 80%-100% | Target preload = 50 kN |
| Three-step path | Three-step tightening | 40%-70%-100%; 40%-80%-100%; 50%-70%-100% | Target preload = 50 kN |

**Appendix Table A2. Bolt-level residual preload values under single-step tightening (B1-B5).**

| Condition | B1 | B2 | B3 | B4 | B5 |
| --- | --- | --- | --- | --- | --- |
| Sequential tightening of the shaft disk | 49488.58 | 49745.29 | 49730.65 | 49726.32 | 49739.03 |
| Single-axis crisscross tightening on the shaft disk | 49486.07 | 49999.41 | 49475.27 | 49734.52 | 49984.29 |
| Dual-axis crisscross tightening on the shaft disk | 49488.53 | 49759.55 | 49740.49 | 49722.1 | 49984.35 |
| Sequential tightening of the curvic coupling | 33275 | 37798 | 39658 | 40699 | 41368 |
| Single-axis crisscross tightening of the curvic coupling | 33017 | 46107 | 35882 | 40673 | 49319 |
| Dual-axis crisscross tightening of the curvic coupling | 33237 | 38280 | 40956 | 43697 | 49315 |

**Appendix Table A2. Bolt-level residual preload values under single-step tightening (B6-B10).**

| Condition | B6 | B7 | B8 | B9 | B10 |
| --- | --- | --- | --- | --- | --- |
| Sequential tightening of the shaft disk | 49756.83 | 49770.35 | 49765.79 | 49747.4 | 50000 |
| Single-axis crisscross tightening on the shaft disk | 49506.25 | 50014.97 | 49492.6 | 49748.76 | 50000 |
| Dual-axis crisscross tightening on the shaft disk | 49508.53 | 49776.52 | 49757.99 | 49736.08 | 50000 |
| Sequential tightening of the curvic coupling | 41901 | 42477 | 43305 | 45075 | 50000 |
| Single-axis crisscross tightening of the curvic coupling | 33637 | 46798 | 36333 | 41277 | 50000 |
| Dual-axis crisscross tightening of the curvic coupling | 33859 | 38874 | 41511 | 44294 | 50000 |

**Appendix Table A3. Bolt-level residual preload values under different initial preload levels (B1-B5).**

| Condition | B1 | B2 | B3 | B4 | B5 |
| --- | --- | --- | --- | --- | --- |
| Shaft disk 30 kN | 29694.79 | 29856.16 | 29845.08 | 29834.11 | 29990.66 |
| Shaft disk 40 kN | 39590.94 | 39807 | 39792.42 | 39777.79 | 39987.48 |
| Shaft disk 50 kN | 49488.53 | 49759.55 | 49740.49 | 49722.1 | 49984.35 |
| Shaft disk 60 kN | 59386.22 | 59710.65 | 59688.57 | 59666.49 | 59981.22 |
| Curvic coupling 30 kN | 19931 | 22959 | 24565 | 26211 | 29588 |
| Curvic coupling 40 kN | 26582 | 30620 | 32762 | 34954 | 39452 |
| Curvic coupling 50 kN | 33237 | 38280 | 40956 | 43697 | 49315 |
| Curvic coupling 60 kN | 39893.12 | 45942.13 | 49151.38 | 52438.52 | 59178.44 |

**Appendix Table A3. Bolt-level residual preload values under different initial preload levels (B6-B10).**

| Condition | B6 | B7 | B8 | B9 | B10 |
| --- | --- | --- | --- | --- | --- |
| Shaft disk 30 kN | 29705.82 | 29866.88 | 29855.48 | 29843.96 | 30000 |
| Shaft disk 40 kN | 39606.64 | 39821.35 | 39806.36 | 39791.13 | 40000 |
| Shaft disk 50 kN | 49508.53 | 49776.52 | 49757.99 | 49736.08 | 50000 |
| Shaft disk 60 kN | 59410.14 | 59731.82 | 59709.52 | 59686.55 | 60000 |
| Curvic coupling 30 kN | 20306 | 23319 | 24901 | 26572 | 30000 |
| Curvic coupling 40 kN | 27082 | 31096 | 33205 | 35434 | 40000 |
| Curvic coupling 50 kN | 33859 | 38874 | 41511 | 44294 | 50000 |
| Curvic coupling 60 kN | 40638.29 | 46654.96 | 49817.21 | 53157.88 | 60000 |

**Appendix Table A4. Bolt-level residual preload values under two-step tightening (B1-B5).**

| Condition | B1 | B2 | B3 | B4 | B5 |
| --- | --- | --- | --- | --- | --- |
| Shaft disk20% | 49590.69 | 49801.62 | 49792.04 | 49779.6 | 49988.17 |
| Curvic coupling 20% | 36036 | 40226 | 42525 | 44862 | 49445.41 |
| Shaft disk40% | 49692.61 | 49850.26 | 49842.94 | 49834.75 | 49990.92 |
| Curvic coupling 40% | 38892.79 | 42236.63 | 44124.07 | 46040.59 | 49575.8 |
| Shaft disk60% | 49794.74 | 49899.63 | 49894.56 | 49889.6 | 49993.46 |
| Curvic coupling 60% | 41745.63 | 44262.14 | 45732.12 | 47223.43 | 49703.25 |
| Shaft disk 80% | 49896.86 | 49949.17 | 49946.5 | 49944.61 | 49996.73 |
| Curvic coupling 80% | 44595.21 | 46291.4 | 47341.26 | 48397.15 | 49833.57 |

**Appendix Table A4. Bolt-level residual preload values under two-step tightening (B6-B10).**

| Condition | B6 | B7 | B8 | B9 | B10 |
| --- | --- | --- | --- | --- | --- |
| Shaft disk20% | 49602.57 | 49814.82 | 49805.59 | 49792.71 | 50000 |
| Curvic coupling 20% | 36517 | 40715 | 42300 | 45367.58 | 50000 |
| Shaft disk40% | 49701.75 | 49860.33 | 49852.92 | 49844.09 | 50000 |
| Curvic coupling 40% | 39283.97 | 42632.27 | 44516.99 | 46452.07 | 50000 |
| Shaft disk60% | 49801.1 | 49906.45 | 49901.2 | 49895.77 | 50000 |
| Curvic coupling 60% | 42060.15 | 44578.43 | 46042.08 | 47535.84 | 50000 |
| Shaft disk 80% | 49900.1 | 49952.7 | 49949.91 | 49947.83 | 50000 |
| Curvic coupling 80% | 44830.17 | 46528.33 | 47571.71 | 48617.88 | 50000 |

**Appendix Table A5. Bolt-level residual preload values under three-step tightening (B1-B5).**

| Condition | B1 | B2 | B3 | B4 | B5 |
| --- | --- | --- | --- | --- | --- |
| Shaft disk40%-70%-100% | 49845.58 | 49924.46 | 49920.68 | 49916.71 | 49995.06 |
| curvic coupling40%-70%-100% | 44062.16 | 45872.92 | 46917.54 | 47960.44 | 49780.89 |
| Shaft disk40%-80%-100% | 49896.84 | 49949.41 | 49946.84 | 49944.42 | 49996.71 |
| curvic coupling40%-80%-100% | 45485.02 | 46886.59 | 47721.75 | 48546.33 | 49845.96 |
| Shaft disk50%-70%-100% | 49845.72 | 49924.59 | 49920.83 | 49916.73 | 49995.06 |
| curvic coupling50%-70%-100% | 44287.39 | 46023.28 | 47013.43 | 47999.18 | 49784.01 |

**Appendix Table A5. Bolt-level residual preload values under three-step tightening (B6-B10).**

| Condition | B6 | B7 | B8 | B9 | B10 |
| --- | --- | --- | --- | --- | --- |
| Shaft disk40%-70%-100% | 49850.31 | 49929.69 | 49925.7 | 49918.68 | 50000 |
| curvic coupling40%-70%-100% | 44283.71 | 46096.23 | 47135.39 | 48182.83 | 50000 |
| Shaft disk40%-80%-100% | 49900.09 | 49952.9 | 49950.23 | 49947.64 | 50000 |
| curvic coupling40%-80%-100% | 45667.91 | 47072.85 | 47899.8 | 48723.05 | 50000 |
| Shaft disk50%-70%-100% | 49850.44 | 49929.8 | 49925.83 | 49918.68 | 50000 |
| curvic coupling50%-70%-100% | 44496.35 | 46233.18 | 47217.92 | 48209.59 | 50000 |

**Appendix Table A6. Friction-coefficient verification summary.**

| Part | Friction coefficient | Mean residual preload (kN) | Minimum residual preload (kN) | Standard deviation (N) | Normalized residual-preload ratio range |
| --- | --- | --- | --- | --- | --- |
| Curvic coupling | 0.1 | 41.2473 | 32.9505 | 5770.32 | 0.41518 |
| Shaft disk | 0.1 | 49.7474 | 49.4885 | 165.3125 | 0.01028 |
| Curvic coupling | 0.15 | 41.4023 | 33.237 | 5677.29 | 0.40654 |
| Shaft disk | 0.15 | 49.7474 | 49.4885 | 165.3123 | 0.01028 |
| Curvic coupling | 0.2 | 41.5414 | 33.4911 | 5593.72 | 0.39891 |
| Shaft disk | 0.2 | 49.7474 | 49.4885 | 165.3123 | 0.01028 |

**Appendix Table A7. Mesh-independence verification summary.**

| Part | Mesh type | Mean residual preload (kN) | Minimum residual preload (kN) | Standard deviation (N) | Normalized residual-preload ratio range | Note |
| --- | --- | --- | --- | --- | --- | --- |
| Curvic coupling | Coarse mesh | 41.452 | 33.32 | 5644.65 | 0.40401 | Summary statistics from mesh-independence verification. |
| Curvic coupling | Fine mesh | 41.436 | 33.301 | 5654.98 | 0.40465 | Summary statistics from mesh-independence verification. |
| Shaft disk | Coarse mesh | 49.74 | 49.476 | 170 |  | Summary statistics from mesh-independence verification. |
| Shaft disk | Fine mesh | 49.752 | 49.5 | 161.68 |  | Summary statistics from mesh-independence verification. |
